# Computed tomography assessment of temporomandibular joint position and dimensions in patients with class II division 1 and division 2 malocclusions

**DOI:** 10.4317/jced.53524

**Published:** 2017-03-01

**Authors:** Hande Gorucu-Coskuner, Semra Ciger

**Affiliations:** 1 Dr, Department of Orthodontics, Faculty of Dentistry, Hacettepe University, Sıhhıye, 6100, Ankara / Turkey

## Abstract

**Background:**

This study aimed to investigate and compare the positions and dimensions of the temporomandibular joint and its components, respectively, in patients with Class II division 1 and division 2 malocclusions.

**Material and Methods:**

Computed tomography images of 14 patients with Class II division 1 and 14 patients with Class II division 2 malocclusion were included with a mean age of 11.4 ± 1.2 years. The following temporomandibular joint measurements were made with OsiriX medical imaging software program. From the sagittal images, the anterior, superior, and posterior joint spaces and the mandibular fossa depths were measured. From the axial images, the greatest anteroposterior and mediolateral diameters of the mandibular condyles, angles between the long axis of the mandibular condyle and midsagittal plane, and vertical distances from the geometric centers of the condyles to midsagittal plane were measured. The independent samples t-test was used for comparing the measurements between the two sides and between the Class II division 1 and 2 groups.

**Results:**

No statistically significant differences were observed between the right and left temporomandibular joints; therefore, the data were pooled. There were statistically significant differences between the Class II division 1 and 2 groups with regard to mandibular fossa depth and anterior joint space measurements.

**Conclusions:**

In Class II patients, the right and left temporomandibular joints were symmetrical. In the Class II division 1 group, the anterior joint space was wider than that in Class II division 2 group, and the mandibular fossa was deeper and wider in the Class II division 1 group.

** Key words:**Temporomandibular joint, Class II malocclusion, Cone beam computed tomography.

## Introduction

Visualizing the temporomandibular joint is difficult using only conventional radiography. Therefore, the position of the condyle in the articular fossa could not be clearly evaluated until the advent of computed tomography (CT) (1). Because of the high radiation dose associated with CT, three-dimensional (3D) assessment was not widespread until the introduction of cone-beam CT (CBCT). Using CBCT, 3D images can be produced at lower radiation doses and decreased cost (2).

The evidence that currently exists regarding the correlation between occlusion and the temporomandibular joint is contradictory. Myers *et al.* (3), Mongini (4), Mongini and Schmid (5), O’Byrn *et al.* (6), and Schudy (7) showed an association between the mandibular fossa-condyle relationship and occlusion; however, Cohlmia *et al.* (8) did not support those findings. When creating an ideal occlusion with orthodontic treatment, the temporomandibular joint position should not be underestimated. If the correlation between the temporomandibular joint position and the occlusion is as important as several orthodontists believe, the condylar position in the mandibular fossa in different types of malocclusion should be clearly evaluated. Although each patient has varying malocclusion characteristics, it can be helpful to visualize the condylar positions in different types of malocclusion. There are a few studies comparing condylar positions in different types of malocclusion (8-10); however, to the best of our knowledge, only a few of those studies used CBCT for the evaluation (11).

Because there is an absence of scientific evidence for coronal joint measurements of the temporomandibular joint (12), we aimed to investigate and compare positions and dimensions of the temporomandibular joint and its components, respectively, in patients with Class II division 1 and division 2 malocclusions.

## Material and Methods

The data used for this study were selected from the archive of Hacettepe University Faculty of Dentistry Department of Orthodontics. 14 patients with Class II division 1 malocclusion (group I) and 14 patients with Class II division 2 malocclusion (group II) who had undergone CBCT for a previously published prospective clinical trial (13) were selected for this present study. The ethical approval was granted by the Hacettepe University Ethical Committee (institutional review board number: GO 16/25-19). Group I included 8 girls and 6 boys, and Group II included 6 girls and 8 boys. The patients’ age range was between 9-13 years, with a mean of 11.4 ± 1.2 years.

The inclusion criteria for Class II division 1 group were:

• to have a Class II division 1 malocclusion with mandibular retrusion (SNA < 82°, SNB < 78°, ANB > 3,5°), 

• to have an overjet > 5 mm,

• to have a Class II or end-to-end molar relationship, 

• to be in the pubertal growth period according to the cervical vertebral maturation method.

The inclusion criteria for Class II division 2 group were:

• to have a Class II division 2 malocclusion with mandibular retrusion (SNA<82°, SNB<78°, ANB>3,5°), 

• to have an overbite > 3,5 mm,

• to have palatally inclined upper incisors (U1-FH < 111°),

• to have a Class II or end-to-end molar relationship, 

• to be in the pubertal growth period according to the cervical vertebral maturation method.

The exclusion criteria for both groups were:

• history of facial trauma, 

• temporomandibular disorders, 

• cross-bite, 

• functional mandibular deviation,

• facial asymmetry.

CBCT was performed using the Iluma Cone Beam CT Scanner (3M IMTEC, Ardmore, OK, USA) at 3.8 mA, 120 kVp, and a 19 × 24 field of view. Patients were seated in a natural head posture for maximum dental intercuspation. The cephalometric tracings and measurements were performed using the Quick Ceph program (Quick Ceph System 2012, San Diego, CA, USA). The following cephalometric measurements were obtained for determining the groups: sella-nasion-A point angle (SNA°), sella-nasion-B point angle (SNB°), A point-nasion-B point angle (ANB°), Frankfurt horizontal- mandibular plane angle (FMA°), lower facial height angle (LFH°), upper incisor- Frankfurt horizontal angle (U1-FH°), overjet (mm), and overbite (mm). For temporomandibular joint measurements, the CBCT images were saved as DICOM files, and processed using the OsiriX medical imaging software program (Open-Source, OsiriX Medical Imaging Software, www.osirix-viewer.com), as previously described by Leonardi *et al.* (14) The long axis, which was defined as the line passing through the midline of the condyle in the coronal and axial sections was determined and the sagittal image was constructed.

The following linear measurements were obtained from the sagittal image: 1) the anterior joint space (the shortest distance between the most anterior point of the condyle and posterior wall of the articular tubercle), 2) the superior joint space (the shortest distance between the most superior point of the condyle and deepest point of the mandibular fossa), 3) the posterior joint space (the shortest distance between the most posterior point of the condyle and the posterior wall of the mandibular fossa), and 4) the depth of the mandibular fossa (the distance between the deepest point of the mandibular fossa and the plane formed by the most inferior point of the articular tubercle to the most inferior point of the auditory meatus).

Further, the midsagittal plane was determined in the coronal and sagittal sections as a plane perpendicular to the line from the anterior nasal spine to the posterior nasal spine (ANS-PNS) and an axial image was constructed. The following linear measurements were obtained from the axial image: 1) the greatest anteroposterior diameter of the mandibular condyle, 2) the greatest mediolateral diameter of the mandibular condyle, 3) the angle between the long axis of the mandibular condyle and the midsagittal plane, and 4) the vertical distance from the geometric centers of the condyles to the midsagittal plane.

-Statistical Analyses.

Every measurement was obtained twice by the same blinded observer with a 3- week interval between the first and second measurements. To assess the reproducibility of all the measurements, intraclass correlation coefficients (ICC) and 95% confidence intervals were determined.

The distribution of data was evaluated using the Kolmogorov-Smirnov test. The independent samples t-test was used to evaluate the differences between the groups and within the groups between the right and left temporomandibular joint measurements. There were no statistically significant differences for right and left temporomandibular joint measurements; therefore, the data from the two joints were pooled together. Subsequently, the independent samples t-test was used to compare the measurements between the groups. Descriptive statistics were calculated as mean ± standard deviation. A *p* value of <0.05 was considered to be statistically significant. The data were analyzed using IBM SPSS Statistics Version 21.0.

## Results

The ICC values differed between 0.88 and 0.99. Mean values and standard deviations of the cephalometric measurements are shown in  Table 1. According to the measurements, the patients in both groups had a Class II skeletal relationship because of mandibular retrusion (SNB <78°), and no statistically significant differences were found in the SNA, SNB, and ANB angles between the two groups. Both of the groups showed mainly normodivergent characteristics with respect to FMA with a slight hypodivergence tendency at Group II with respect to LFH. Although the LFH measurements were smaller in group II, there were no statistically significant differences between the groups. The U1-FH angle, overjet, and overbite values showed significant differences between the groups. With regard to the U1-FH angle, the upper incisors were proclined in group I and retroclined in group II. The overjet was significantly excessive in group I and the overbite was significantly excessive in group II.

**Table 1:**
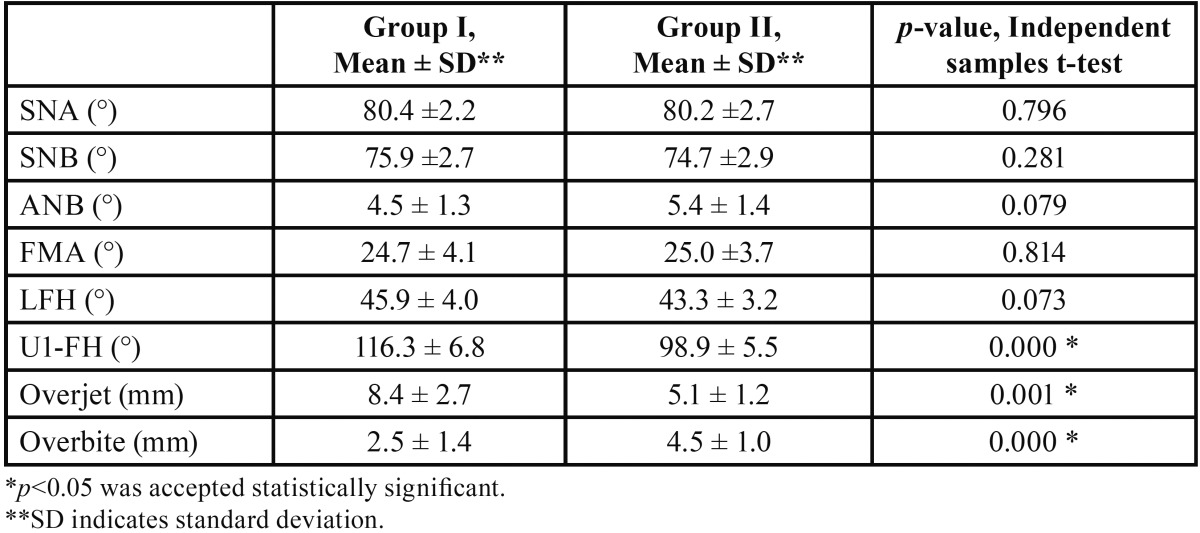
Statistical analysis of the comparison between Class II division 1 and division 2 malocclusion group-cephalometric values.

With regard to the temporomandibular joint measurements, there were no statistically significant differences between the right and left joints; therefore, the two sides were pooled together. Thereby, in both the Class II division 1 and division 2 groups 28 joints were evaluated.

The descriptive statistics for each measurement evaluated in comparison of the two groups and those for the assessment of the concentric position of the condyles in both groups are shown in  Table 2.

**Table 2:**
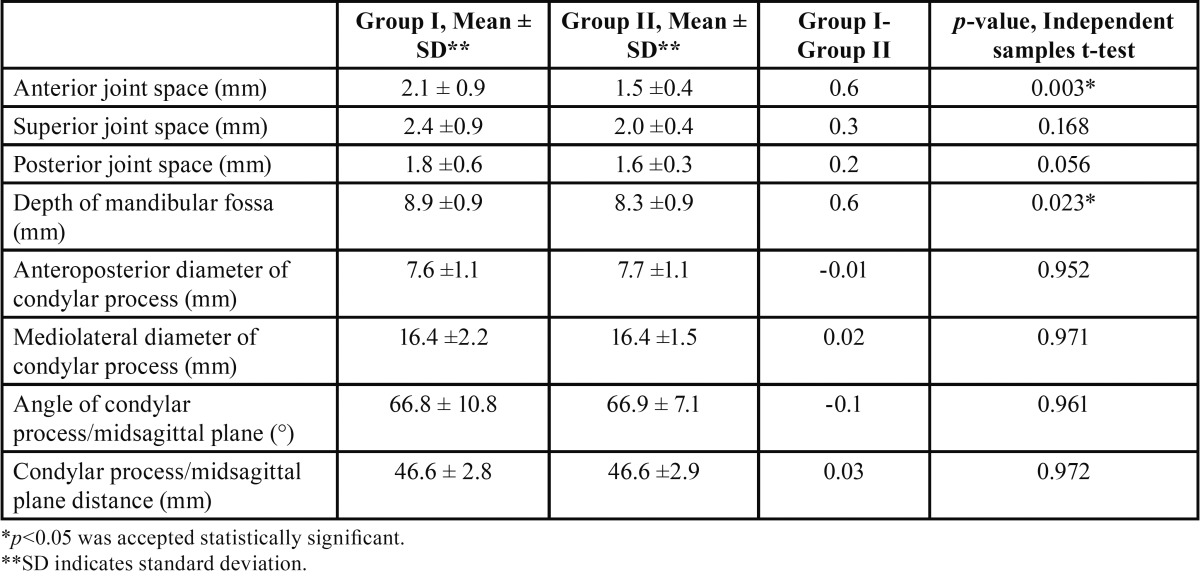
Statistical analysis of the comparison between Class II division 1 and division 2 malocclusion groups-temporomandibular joint values.

The mean anterior joint spaces were 2.1 and 1.5 mm for groups I and II, respectively, and there were statistically significant differences between the groups (*p* = 0.003). The mean superior joint spaces were 2.4 and 2.0 mm for groups I and II, respectively, and there were no statistically significant differences between the groups (*p* = 0.168). The mean posterior joint spaces were 1.8 and 1.6 mm for groups I and II, respectively, and there were no statistically significant differences between the groups (*p* = 0.056). The mean depths of the mandibular fossa were 8.9 and 8.3 mm for groups I and II, respectively, and there were statistically significant differences between the groups (*p* = 0.023).

The mean values for the anteroposterior diameter of the condylar processes were 7.6 and 7.7 mm for groups I and II, respectively, and there were no statistically significant differences between the groups (*p* = 0.952). The mean values for the mediolateral diameter of the condylar processes were 16.4 mm for both groups, and there were no statistically significant differences between the groups (*p* = 0.971).

The angles between the plane of the greatest mediolateral diameter of the condylar processes and the midsagittal plane were 66.8° and 66.9° for groups I and II, respectively, and there were no statistically significant differences between the groups (*p* = 0.961). The mean values for the distance from the geometric center of the condylar processes to the midsagittal plane were 46.6 mm for both groups, and there were no statistically significant differences between the groups (*p* = 0.972).

In the evaluation of the concentric position of the condyles in group I, the mean values for the anterior and posterior joint spaces were 2.1 and 1.8 mm, respectively, and the difference between the anterior and posterior joint spaces was 0.2 mm. ( Table 3). In group II, the mean values for the anterior and posterior joint spaces were 1.5 and 1.6 mm, respectively, and the difference between the anterior and posterior joint spaces was -0.1 mm. There were no statistically significant differences between the groups for these measurements (*p* = 0.117).

**Table 3:**
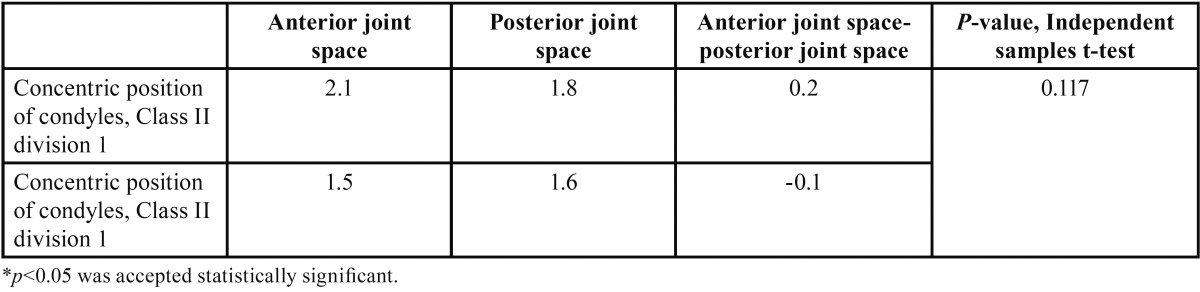
Statistical analysis: concentric position of the condyles.

## Discussion

CT imaging is an ideal tool for evaluating the temporomandibular joint (15). By using 3D imaging, it is possible to eliminate superimposition of other structures and obtain more accurate measurements. Hilgers *et al.* (16) conducted a study comparing CBCT and conventional radiographic measurements of temporomandibular joint images in 25 dry skulls. They concluded that all CBCT measurements were reproducible and significantly more accurate than the measurements from conventional cephalograms.

Patients with posterior crossbites were not included in this present study because such patients may have mandibular deviations. Furthermore, all our patients were evaluated for mandibular deviation because it is known that when mandibular deviation is present, the condyle adapts to the new position of the mandible (8,17) and all temporomandibular joint measurements alternate. Furthermore, patients with signs and symptoms of temporomandibular disorders were not included in this study. However, because magnetic resonance images were not obtained, the disc positions in the patients were unknown; this is a potential limitation of this study.

This study aimed to investigate and compare the positions and dimensions of the temporomandibular joint and its components, respectively, in patients with Class II division 1 and division 2 malocclusions. When examining the results of this study, the malocclusions should be considered to either be the consequence or cause of the variations in temporomandibular joint position.

Both groups were selected as mandibular retrusion patients (SNB <78°) with group I included Class II division 1 patients and group II included Class II division 2 patients. Class II division 1 malocclusion does not have an evident vertical growth pattern (18); however, Class II division 2 malocclusion mainly involves a hypodivergent growth pattern (19). Furthermore, the rotation of the mandible may affect mandibular condyle morphology and position (20). In the present study, the facial pattern of both groups were mainly normodivergent with a hypodivergence tendency at group II. No statistically significant differences were found between the groups. This study aimed to evaluate the differences of Class II division 1 and division 2 malocclusions in general; therefore, for the evaluation of the effects of facial patterns on temporomandibular joint positions, advanced studies require to be conducted with groups that include different facial patterns in patients with Class II division 1 and division 2 malocclusions.

The findings of the present study are of paramount importance because previous studies that have evaluated condylar spaces using CBCT images are limited (11,13,14,21). When the sagittal image was being constructed, the long axis of the condyle was selected as the line passing through the central aspect of the condyle. This was because joint spaces may differ between the medial, central, and lateral aspects. When the measurements are obtained using two-dimensional radiographs, it is impossible to assess the temporomandibular joint at a standart aspect; so 3-dimensional imaging is an important tool for temporomandibular joint evaluation.

In the present study, when the right and left temporomandibular joints were compared within the two groups, there were no statistically significant differences for the anterior, posterior, and superior joint spaces and the mandibular fossa depth. Furthermore, this finding supports the fact that no patient had mandibular deviation. The studies that evaluate condylar symmetry in Class I (22), Class II division 1 subdivision (23), and Class III (15) patients showed symmetrical temporomandibular joint spaces and mandibular fossa depths. In the Class II division 1 group, the patients had no functional mandibular deviation or facial asymmetry, resulting in symmetrical joint spaces. However, Cohlmia *et al.* (8) found that the left condyle was positioned more anteriorly than the right in all malocclusions. Moreover, Rodrigues *et al.* (15) found a statistically significant difference between the right and the left posterior joint spaces in Class II division 1 patients.

Ricketts (24) stated that in Class II division 1 patients, the condyles were more anteriorly positioned, and suggested that this may be present to relieve the narrower airways in such patients. Pullinger *et al.* (10) evaluated temporomandibular joint tomograms in 44 adult patients and concluded that in Class II division 1 patients, the condyles were more anteriorly positioned than in Class I patients. Cohlmia *et al.* (8) found no significant difference between Class I and Class II patients. Fang and Tao (25) evaluated the condyle- fossa relationships in Class I and Class II division 1 patients and concluded that in Class II division 1 patients, the anterior joint space was decreased and the posterior joint space was increased compared with Class I patients.

Our results showed no significant difference between the groups for posterior and superior joint spaces. However, the anterior joint space was significantly narrower in the Class II division 2 patients than in the Class II division 1 patients. Because there were no statistically significant differences found in condylar dimensions and concentricity between the two groups, the difference in the anterior joint space may be explained by different dimensions of the mandibular fossa, as Rodrigues *et al.* previously stated (15,22).

Ikeda and Kawamura (11) assessed temporomandibular joint positions in 22 patients with optimal joints to determine the mean anterior, superior, and posterior joint spaces in healthy joints. They reported that the optimal anterior, superior, and posterior joint spaces as being 1.3 ± 0.2, 2.5 ± 0.5, and 2.1 ± 0.3 mm, respectively. Further studies should be conducted with larger sample sizes to determine and compare the joint spaces of Class I, Class II divisions 1 and 2, and Class III patients to further the understanding of optimal joint spaces and the variations in different malocclusions.

In this study, the mandibular fossa was deeper in the Class II division 1 group than in the Class II division 2 group. Mandibular fossa depth is known to be highly affected by anterior guidance (26-28). In our study, open-bite patients were not included because such patients may have significantly smaller vertical heights of the mandibular fossa (8); however, there was no limitation in the patient selection regarding the depth of the bite. Therefore, it would be deceptive to associate the mandibular fossa depth only with the type of malocclusion.

We found no statistically significant differences for the anteroposterior and mediolateral dimensions of the condyles between the right and the left sides in both groups. This is in agreement with other studies that evaluated symmetry in different types of malocclusions (15,22,23). Also, there were no statistically significant differences between the groups for the anteroposterior and mediolateral dimensions of the condyles. To the best of our knowledge, no other studies have compared the anteroposterior and mediolateral dimensions of the condyles between patients with Class II division 1 and division 2 malocclusions. However, Katsavrias and Halazonetis (9) compared the condyle and fossa shapes of these malocclusions and found no statistically significant differences.

With regard to the assessment of the distance of condylar process/midsagittal plane and angulation of the condylar processes associated with the midsagittal plane, there were no significant differences between the right and left sides. Condyle positional asymmetry is most frequently associated with functional deviations, and, because there were no functional deviations in our groups, this finding was expected and consistent with most of the previous studies (15,22,23). Furthermore, there were no statistically significant differences between the groups for the aforementioned measurements. This finding supports the fact that in patients with Class II division 1 and division 2 malocclusions, condylar position and shape in the axial plane are similar.

Evaluation of the condylar concentricity showed that in both malocclusion types, the condyles were nonconcentrically positioned. Although in the Class II division 1 group, the condyles appeared to be more posteriorly positioned than in the Class II division 2 group, there were no statistically significant differences between the groups.

Katsavrias and Halazonetis (9) stated that in patients with Class II division 1 malocclusion, the condyles were more anteriorly positioned than in those with Class II division 2 malocclusion. Rodrigues *et al.* (15) evaluated condylar concentricity in Class II division 1 patients and concluded that the condyles were more anteriorly positioned.

From the literature, the difference in the condylar positioning in our groups may be because of the age range of our patients. In our study, the age range was 9-13 years, with a mean of 11.4 ± 1.2 years, rather than a wider pool of patients with regard to the age range. Katsavrias (29) evaluated patients with Class II division 2 malocclusion in different age groups and concluded that the condylar position changes from the anterior to posterior position with age. To evaluate condylar positional changes in Class II division 1 patients, more studies require to be conducted with different age ranges.

Currently, temporomandibular joint oriented treatment planning is considering as one of the main factors for the health of surrounding structures of the condyle and retention of the treatment results. To understand the characteristics and aetiology of Class II malocclusions, and treatment effects to the temporomandibular joint, firstly condylar anatomy and characteristics should be clearly understood. For determination temporomandibular joint characteristics of different malocclusions, further studies should be conducted with the aid of 3 dimensional imaging.

## Conclusions

There were no statistically significant differences between the right and left sides with regard to the condyle-fossa relationship, the depth of mandibular fossa, anteroposterior and mediolateral dimensions of the condyles, the distance of the condylar process to the midsagittal plane, and the angulation of the condylar processes associated with the midsagittal plane within the groups.

In the Class II division 1 group, the anterior joint space was wider than that in the Class II division 2 group, and the mandibular fossa was deeper and wider in the Class II division 1 group.

There were no statistically significant differences between the groups for superior and posterior joint spaces, anteroposterior and mediolateral dimensions of the condyles, the distance of the condylar process to the midsagittal plane, and the angulation of the condylar processes with regard to the midsagittal plane within the groups.
